# Interplay between Thyroid Hormones and Stearoyl-CoA Desaturase 1 in the Regulation of Lipid Metabolism in the Heart

**DOI:** 10.3390/ijms22010109

**Published:** 2020-12-24

**Authors:** Adam Olichwier, Volodymyr V. Balatskyi, Marcin Wolosiewicz, James M. Ntambi, Pawel Dobrzyn

**Affiliations:** 1 Laboratory of Molecular Medical Biochemistry, Nencki Institute of Experimental Biology, Polish Academy of Sciences, 02-093 Warsaw, Poland; a.olichwier@nencki.edu.pl (A.O.); v.balatskyi@nencki.edu.pl (V.V.B.); m.wolosiewicz@nencki.edu.pl (M.W.); 2 Department of Biochemistry, University of Wisconsin-Madison, Madison, WI 53706, USA; jmntambi@wisc.edu; 3 Department of Nutritional Sciences, University of Wisconsin-Madison, Madison, WI 53706, USA

**Keywords:** SCD1, lipogenesis, lipolysis, β-oxidation, hypothyroidism, inflammation

## Abstract

Stearoyl-CoA desaturase 1 (SCD1), an enzyme that is involved in the biosynthesis of monounsaturated fatty acids, induces the reprogramming of cardiomyocyte metabolism. Thyroid hormones (THs) activate both lipolysis and lipogenesis. Many genes that are involved in lipid metabolism, including *Scd1*, are regulated by THs. The present study used SCD1 knockout (SCD1^−/−^) mice to test the hypothesis that THs are important factors that mediate the anti-steatotic effect of SCD1 downregulation in the heart. SCD1 deficiency decreased plasma levels of thyroid-stimulating hormone and thyroxine and the expression of genes that regulate intracellular TH levels (i.e., *Slc16a2* and *Dio1-3*) in cardiomyocytes. Both hypothyroidism and SCD1 deficiency affected genomic and non-genomic TH pathways in the heart. SCD1 deficiency is known to protect mice from genetic- or diet-induced obesity and decrease lipid content in the heart. Interestingly, hypothyroidism increased body adiposity and triglyceride and diacylglycerol levels in the heart in SCD1^−/−^ mice. The accumulation of triglycerides in cardiomyocytes in SCD1^−/−^ hypothyroid mice was caused by the activation of lipogenesis, which likely exceeded the upregulation of lipolysis and fatty acid oxidation. Lipid accumulation was also observed in the heart in wildtype hypothyroid mice compared with wildtype control mice, but this process was related to a reduction of triglyceride lipolysis and fatty acid oxidation. We also found that simultaneous SCD1 and deiodinase inhibition increased triglyceride content in HL-1 cardiomyocytes, and this process was related to the downregulation of lipolysis. Altogether, the present results suggest that THs are an important part of the mechanism of SCD1 in cardiac lipid utilization and may be involved in the upregulation of energetic metabolism that is associated with SCD1 deficiency.

## 1. Introduction

The heart is a main target of the actions of thyroid hormones (THs). Changes in cardiac function and metabolism are important components of the clinical implications of hypothyroidism (i.e., low TH levels) and hyperthyroidism (i.e., high TH levels; [1]). Levels of THs are linked to the regulation of body weight and energy expenditure. Hyperthyroidism promotes a hypermetabolic phenotype, whereas hypothyroidism is linked with hypometabolism [2]. Deiodinases (DIOs) are a major family of enzymes that regulate TH tissue content because they change triiodothyronine (T3) level removing specific iodine atoms form its precursor thyroxine (T4; [3]). The functional effects of THs are mediated by interactions with TH receptors (TRs) in specific regions of target genes, by which they regulate gene expression. Moreover, THs mediate non-genomic effects that indirectly modulate numerous signaling pathways through the activation of kinases and other transcription factors [2].

Thyroid hormones regulate the expression of numerous genes that are engaged in lipogenesis (e.g., liver X receptors [4] and carbohydrate-responsive element-binding protein [5]) by binding to TRs [6]. Furthermore, THs indirectly control the transcriptional regulation of lipogenesis as a consequence of their effects on the expression and activity of other transcription factors, such as sterol regulatory element-binding protein 1c (SREBP1c), that play a crucial role in lipogenesis [6,7]. TH also change the expression of genes that are involved in lipogenesis in rodents, such as fatty acid synthase (FAS), in a non-genomic manner [8]. Moreover, THs reduce the activity of glycerol-3-phosphate acyltransferase 3 (GPAT3), which is required for triglyceride (TG) synthesis [9].

Although THs increase the expression of genes that are involved in de novo lipogenesis, it does not lead to a net increase in levels of TGs in mice liver [10]. The main reason for this lack of an elevation is a higher rate of fatty acid (FA) uptake [11] and oxidation and an increase in lipolysis [12,13]. Thyroid hormone-induced lipogenesis was suggested to primarily maintain fat loss that occurs through TH-induced lipolysis [12]. Interestingly, in hypothyroidism, lower rates of whole-body energy metabolism result in lower lipid oxidation, except no changes in adipose tissue lipolysis [14]. In the heart, the relationship between TH-dependent lipogenesis and lipolysis/FA oxidation is not fully understood, although increased (by 20%) cardiac lipid content was observed in hypothyroid human subjects [15].

Stearoyl-CoA desaturase (SCD) is a rate-limiting enzyme that catalyzes the synthesis of monounsaturated fatty acids (FAs), mainly oleate and palmitoleate, from saturated fatty acyl-CoAs [16]. Mice with deletion of the *Scd1* gene exhibit higher energy expenditure, elevated basal thermogenesis, decreased body adiposity, and increased insulin sensitivity and are resistant to diet-induced (e.g., by high-fat and high-carbohydrate diets) and genetic-induced (e.g., by leptin deficiency and in agouti mice) obesity [17,18]. SCD1 was recently shown to be a critical regulatory factor of metabolism and function in the heart [16]. The lack of *Scd1* expression decreases FA uptake and utilization in the heart [19]. Loss of the *Scd1* gene improves cardiac function in obese ob/ob mice [20]. This improvement is related to a downergulation of the expression of genes that are involved in FA transport and lipogenesis in the heart, together with reduction in cardiac free FA (FFA), diacylglycerol (DAG), TG, and ceramide levels and a decrease in cardiomyocyte apoptosis [20]. SCD1 deficiency or inhibition reduces cardiac lipid content independently of the action of peroxisome proliferator-activated receptor α (PPARα) by decreasing lipogenesis and elevating lipolysis [21]. Waters et al. [22] reported that T3 administration decreased *Scd1* mRNA levels in the hypothyroid mouse liver and identified a negative TH-response region in the mouse *Scd1* gene promoter. Hashimoto et al. [23] found that TH negatively regulated human *Scd1* gene expression without the direct binding of TRs to the *Scd1* gene promoter. Inoue et al. [24] and Li et al. [25] reported that unesterified long-chain FAs and long-chain fatty acyl-CoAs inhibited TH binding to nuclear receptors. Both studies established that oleic acid (i.e., the product of the SCD1 reaction) was the most potent inhibitor. Moreover, SCD1 reaction substrates (i.e., palmitate, stearate, and their acyl-CoAs) also resulted in a strong reduction of the receptors’ affinity for T3 [24,25]. Furthermore, an association of one SCD single nucleotide polymorphism with Graves’ ophthalmopathy but not with Graves’ disease, an immune system disorder that results in the overproduction of thyroid hormones, was demonstrated [26].

Both SCD1 and THs play an important role in regulating cardiac metabolism and function. In the present study, we investigated the possible interaction between SCD1 and THs in the regulation of lipid metabolism in the heart. Our data showed that under basal conditions, the loss of SCD1 decreased plasma levels of thyroid-stimulating hormone (TSH) and T4, indicating a hyperthyroidic state in these mice. SCD1 ablation also affected genomic and non-genomic TH pathways and reduced inflammatory protein levels in the heart in mice with hypothyroidism. We also found that hypothyroidism increased TG content in the heart in SCD1-deficient (SCD1^−/−^) mice. This phenomenon was associated with the activation of lipogenesis, which surpassed lipolysis and FA oxidation. The present data suggest that SCD1 is an important component of TH turnover and action in the heart and that normal TH metabolism is necessary to maintain the anti-steatotic effect of SCD1 downregulation in the heart.

## 2. Results

### 2.1. Hypothyroidism Decreases Heart Weight and Increases Adiposity in Wildtype and SCD1^−/−^ Mice

Male wildtype (WT) and SCD1^−/−^ mice were fed for 7 weeks a low-iodine diet supplemented with 0.15% propylthiouracil to induce hypothyroidism or standard laboratory chow. Hypothyroidism increased white adipose tissue (WAT) mass and decreased the heart weight/body weight ratio in both WT and SCD1^−/−^ mice (Table 1). Plasma TG levels significantly decreased in all groups compared with WT controls, whereas FFA levels were lower in the WT hypothyroid (Hypo) group than in WT controls. Hypothyroidism increased plasma glucose levels in both WT and SCD1^−/−^ mice (Table 1). Basal plasma levels of TSH and T4 were lower in SCD1^−/−^ mice compared with WT mice. After 7 weeks of the non-iodine diet, TSH levels significantly increased in WT and SCD1^−/−^ mice (2-fold and 14-fold, respectively), and T4 levels decreased (81% and 64%, respectively), whereas free T3 (fT3) levels were unchanged (Table 1).

### 2.2. SCD1 Deficiency Affects Genomic and Non-Genomic TH Pathways in the Heart

**Figure 1:**
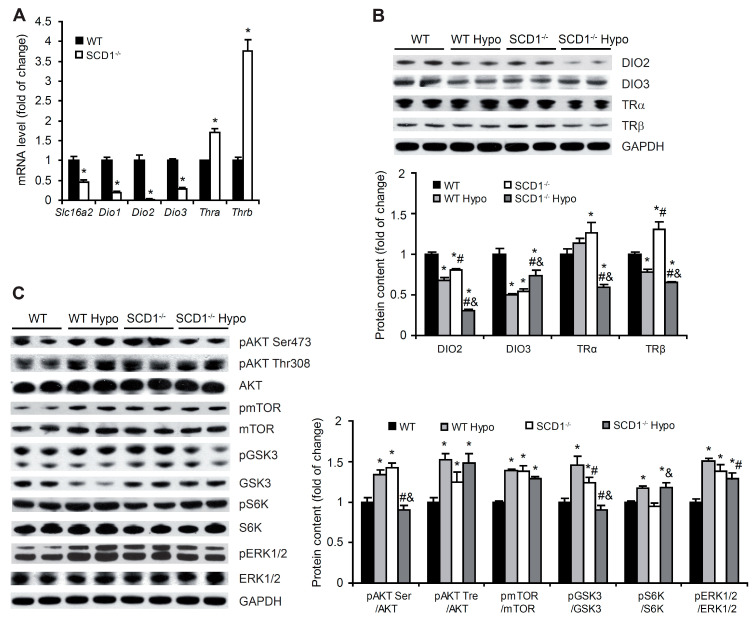
(**A**) Effect of SCD1 deficiency on the expression of genes that are involved in thyroid hormone actions. mRNA levels of *Slc16a2*, *Dio1*, *Dio2*, *Dio3*, *Thra*, and *Thrb* were determined in the heart in wildtype (WT) and SCD1^−/−^ mice by real-time polymerase chain reaction. (**B**,**C**) The levels of proteins that are involved in (**B**) genomic and (**C**) non-genomic thyroid hormone actions in the heart in control and hypothyroid mice were determined by Western blot. AKT, protein kinase B; mTOR, mechanistic/mammalian target of rapamycin; GSK3, glycogen synthase kinase 3; S6K, S6 kinase; ERK1/2, extracellular signal-regulated kinases 1/2; DIO, deiodinase; TR, thyroid receptor. The data are representative of *n* = 5 mice/group. The data are expressed as mean ± SD. * *p* < 0.05, vs. WT; ^#^ *p* < 0.05, vs. WT Hypo; ^&^ *p* < 0.05, vs. SCD1^−/−^.

Monocarboxylate transporter 8 (MCT8) enables cellular influx and the efflux of T4 and T3 in the heart [27]. T4 is in the peripheral tissue converted to T3 to become biologically active and bind TRs. This conversion is regulated by the interplay of DIO enzymes [2]. SCD1 deletion significantly decreased Slc16a2 (which encodes MCT8 protein), Dio1, Dio2, and Dio3 expression but increased Thra and Thrb expression in the mouse heart (Figure 1A). Hypothyroidism decreased protein levels of DIO2, DIO3, and TRβ in cardiomyocytes in WT mice (Figure 1B). In SCD1^−/−^ Hypo mice, DIO2, TRα and TRβ protein levels decreased, whereas DIO3 protein levels increased compared with SCD1^−/−^ control mice (Figure 1B).

The actions of THs can also be indirectly triggered by non-genomic actions by modulating gene transcription through the activation of kinase pathways [2]. Therefore, we measured protein and phosphorylation levels of protein kinase B (AKT), glycogen synthase kinase 3 (GSK3), mechanistic/mammalian target of rapamycin (mTOR), S6 kinase (S6K), and extracellular signal-regulated kinase 1/2 (ERK1/2) in the heart in WT and SCD1^−/−^ mice by Western blot. The loss of SCD1 increased phosphorylation levels of AKT, GSK3, mTOR, and ERK1/2, whereas S6K phosphorylation was unaffected by SCD1 deficiency (Figure 1C). Hypothyroidism led to the activation of AKT, GSK3, mTOR, S6K, and ERK1/2 in the heart in WT mice. SCD1 deficiency in hypothyroid mice did not affect the phosphorylation of AKT at Thr308, mTOR, or ERK1/2, whereas the phosphorylation of AKT at Ser473 and GSK3 decreased (Figure 1C). S6K phosphorylation increased by 25% in SCD1^−/−^ Hypo mice compared with SCD1^−/−^ controls (Figure 1C). These results indicate that SCD1 expression plays a significant role in the control of genomic and non-genomic pathways of THs in the heart.

### 2.3. Effect of SCD1 Deficiency and Hypothyroidism on Inflammatory Factor Content in the Heart

**Figure 2:**
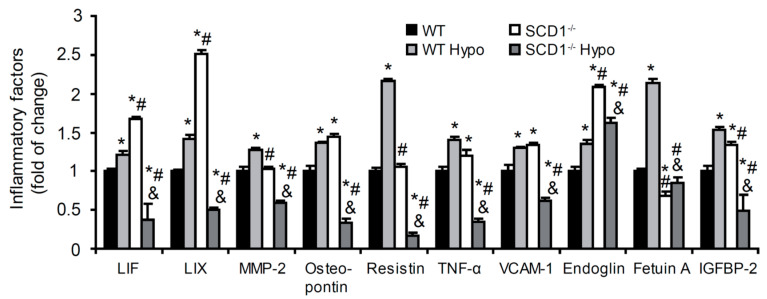
Effect of SCD1 deficiency on the protein content of inflammatory molecules in cardiomyocytes in control and hypothyroid mice. Cytokines and chemokines were measured using the Proteome Profiler Mouse Cytokine Array kit (R&D Systems) according to the manufacturer’s procedures. LIF, leukemia inhibitory factor; LIX, C-X-C motif chemokine 5; MMP-2, matrix metalloproteinase 2; TNF-α, tumor necrosis factor α; VCAM-1, vascular cell adhesion molecule 1; IGFBP-2, insulin-like growth factor-binding protein-2. The data are representative of *n* = 5 animals/group. The data are expressed as mean ± SD. * *p* < 0.05, vs. WT; ^#^ *p* < 0.05, vs. WT Hypo; ^&^ *p* < 0.05, vs. SCD1^−/−^.

Thyroid hormones exert direct effects on cardiovascular metabolism by regulating lipid metabolism and the modulation of inflammatory pathways [2]. SCD1 is known to modulate cellular inflammation and stress [18]. To investigate the role of SCD1 and THs in regulating the inflammation process, we measured levels of anti- and proinflammatory molecules in the heart in WT and SCD1^−/−^ mice. The loss of SCD1 expression increased the protein content of leukemia inhibitory factor (LIF), C-X-C motif chemokine 5 (LIX), osteoponin, tumor necrosis factor α (TNF-α), vascular cell adhesion molecule 1 (VCAM-1), endoglin, and insulin-like growth factor-binding protein 2 (IGFBP-2). SCD1 deficiency did not change the levels of matrix metalloproteinase 2 (MMP-2) or resistin, whereas fetuin A levels decreased (Figure 2). Hypothyroidism increased all of the studied factors in the heart in WT mice. The opposite effect was found in the heart in SCD1^−/−^ mice, where the content of inflammatory factors decreased, with the exception of an increase in fetuin A (Figure 2). These results demonstrate the opposing roles of SCD1 in the regulation of inflammatory processes under normal and hypothyroid conditions.

### 2.4. Loss of SCD1 Exacerbates Hypothyroidism Induced Cardiac Steatosis

**Figure 3:**
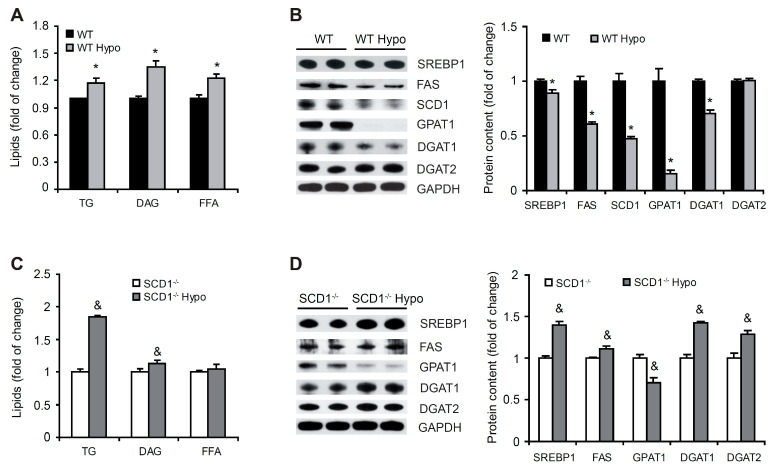
(**A**,**C**) Free fatty acid (FFA), diacylglycerol (DAG), and triglyceride (TG) content in the heart in wildtype (WT) and SCD1^−/−^ mice under physiological and hypothyroid conditions. Lipid extracts were separated by thin-layer chromatography. The plate was soaked in a water mixture that contained cupric sulfate and phosphoric acid and charred. Lipids were then quantified by densitometry. (**B**,**D**) Levels of lipogenic proteins in cardiomyocytes in WT and SCD1^−/−^ mice under physiological and hypothyroid conditions. Protein levels of sterol regulatory element-binding protein 1 (SREBP-1), fatty acid synthase (FAS), stearoyl-CoA desaturase 1 (SCD1), glycerol-3-phosphate acyltransferase 1 (GPAT1), diacylglycerol acyltransferase 1 (DGAT1), and DGAT2 were determined by Western blot. The data are representative of *n* = 5 animals/group. The data are expressed as mean ± SD. * *p* < 0.05, vs. WT; ^&^ *p* < 0.05, vs. SCD1^−/−^.

Mice that lack the Scd1 gene are protected from diet-induced obesity and characterized by lower FA synthesis and higher FA oxidation [16,17]. In the present study, we found that hypothyroidism led to WAT accumulation in both WT and SCD1^−/−^ mice. Next, we measured lipid content in the left ventricle. Significant TG, DAG, and FFA accumulation was observed in the heart in WT Hypo mice compared with WT control mice (by 17%, 35%, and 22%, respectively; Figure 3A). However, this process was not associated with higher levels of lipogenic proteins (Figure 3B).

Interestingly, hypothyroidism led to an almost 2-fold increase in cardiac TG accumulation in the heart in SCD1^−/−^ mice. DAG content also significantly increased (by 13%) in the heart in SCD1^−/−^ Hypo mice compared with SCD1^−/−^ control mice, whereas FFA content was similar (Figure 3C). The increases in TG and DAG content in the heart in SCD1^−/−^ Hypo mice were related to an increase in the level of lipogenic proteins (i.e., SREBP1, FAS, diacylglycerol acyltransferase 1 [DGAT1], and DGAT2; Figure 3D). Interestingly, GPAT1 protein levels decreased in both WT Hypo and SCD1^−/−^ Hypo mice compared with the respective controls (Figure 3B,D). Obtained results indicate that the reduction of fat accumulation in the heart related to SCD1 deficiency [19,20,21] is dependent on the TH action.

### 2.5. SCD1 Deficiency Upregulates Lipolysis in Cardiomyocytes in Hypothyroidism

**Figure 4:**
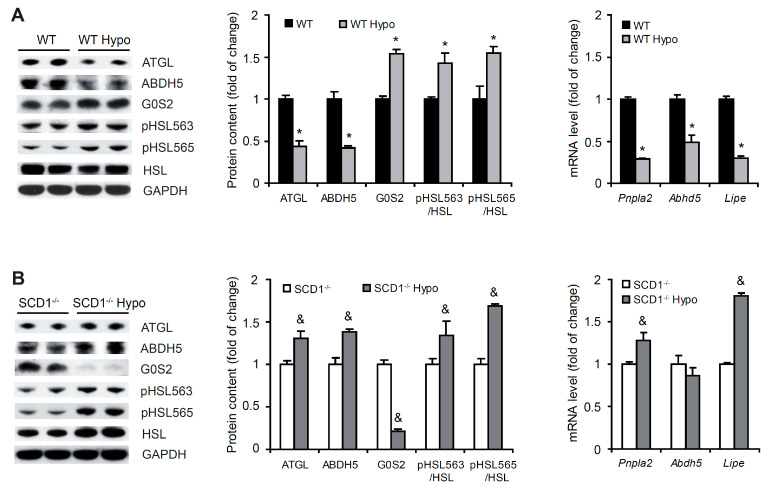
Effect of SCD1 deficiency and hypothyroidism on lipolysis in cardiomyocytes. Protein levels of adipose triglyceride lipase (ATGL), α/β-hydrolase domain containing 5 (ABDH5), G_0_/G_1_ switch protein 2 (G0S2), hormone-sensitive lipase (HSL), and phosphorylated HSL (pHSL) at Ser563 and Ser565 in cardiomyocytes in (**A**) wildtype (WT) and (**B**) SCD1^−/−^ mice were determined by Western blot. mRNA levels of *Pnpla2*, *Abdh5*, and *Lipe* were determined in the heart in WT and SCD1^−/−^ mice by real-time polymerase chain reaction. The data are representative of *n* = 5 animals/group. The data are expressed as mean ± SD. * *p* < 0.05, vs. WT; ^&^ *p* < 0.05, vs. SCD1^−/−^.

Adipose triglyceride lipase (ATGL) catalyzes the rate-limiting step of TG hydrolysis in the heart. G0/G1 switch protein 2 (G0S2) inhibits ATGL, whereas the enzyme is activated by α/β-hydrolase domain containing 5 (ABDH5; [28]). Hypothyroidism decreased the protein content of ATGL and ABDH5, whereas G0S2 content increased in the heart in WT mice (Figure 4A). A similar decrease in the expression of genes that encode ATGL and ABDH5 proteins was observed (Figure 4A). Interestingly, in the heart in SCD1^−/−^ mice, hypothyroidism activated the ATGL pathway, reflected by an increase in the protein content of ATGL and ABDH5 and decrease in the protein content of G0S2. mRNA levels of Pnpla2 (which encodes ATGL protein) increased, whereas mRNA levels of Abdh5 were unaltered in the left ventricle in hypothyroid SCD1^−/−^ mice compared with untreated controls (Figure 4B). These results indicate that the SCD1 ablation increases lipolysis not only in basal condition [21] but also in hypothyroidism.

ATGL generates DAG, which is then hydrolyzed by hormone-sensitive lipase (HSL; [28]) encoded by *Lipe* gene. Protein kinase A (PKA) phosphorylates HSL at Ser563, which activates HSL. In contrast, 5*′*-adenosine monophosphate-activated protein kinase (AMPK) phosphorylates HSL at Ser565, which inhibits HSL activity [29]. The levels of HSL protein and mRNA decreased in cardiomyocytes in WT Hypo mice compared with WT controls (Figure 4A). In contrast, hypothyroidism increased both HSL protein and mRNA levels in SCD1^−/−^ mice (Figure 4B). The phosphorylated HSL at Ser563 (pHSL563)/HSL and at Ser565 (pHSL565)/HSL ratios were higher in hypothyroid WT and SCD1^−/−^ mice compared with the respective control groups (Figure 4A,B).

### 2.6. Higher FA β-Oxidation in the Heart in Hypothyroid SCD1^−/−^ Mice

**Figure 5:**
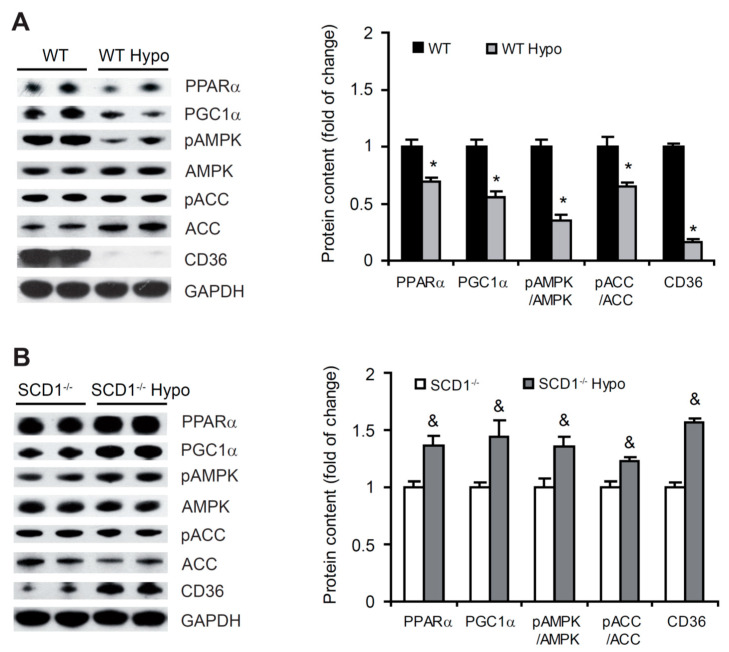
Effect of SCD1 deficiency and hypothyroidism on β-oxidation in the heart. Protein levels of peroxisome proliferator-activated receptor α (PPARα), peroxisome proliferator-activated receptor γ coactivator 1α (PGC1α), 5′-adenosine monophosphate-activated protein kinase (AMPK), phosphorylated AMPK (pAMPK), acetyl-CoA carboxylase (ACC), phosphorylated ACC (pACC), and fatty acid translocase (CD36) in the heart were determined in (**A**) wildtype (WT) and (**B**) SCD1^−/−^ mice by Western blot. The data are representative of *n* = 5 animals/group. The data are expressed as mean ± SD. * *p* < 0.05, vs. WT; ^&^ *p* < 0.05, vs. SCD1^−/−^.

PPARα is a transcription factor that regulates FA catabolism. When activated, PPARα promotes the expression of FA oxidation-related genes [30]. The protein levels of PPARα significantly decreased in the heart in WT Hypo mice compared with WT control mice (Figure 5A). An opposite effect was observed in SCD1^−/−^ mice, in which hypothyroidism caused a significant increase (by 36%) in PPARα protein levels (Figure 5B). To shed light on the molecular mechanism by which PPARα regulates the hyperthyroidic heart, we analyzed the protein levels of peroxisome proliferator-activated receptor γ coactivator 1α (PGC1α), an inducible coregulator of PPARα [30]. Protein levels of PGC1α decreased in WT Hypo mice and increased in SCD1^−/−^ Hypo mice compared with respective controls (Figure 5A,B), indicating that this factor is involved in the regulation of PPARα in the heart in hypothyroidism and that SCD1 deficiency affects PGC1α function.

Fatty acid oxidation is controlled by the activity of AMPK, which is activated by phosphorylation at a threonine residue [31]. We measured AMPK phosphorylation and AMPKα subunit protein levels in the heart in WT and SCD1^−/−^ mice. The loss of SCD1 increased the phosphorylation of AMPK and its downstream target acetyl-CoA carboxylase (ACC) in hypothyroid mice, whereas a decrease in the phosphorylation of AMPK and ACC was observed in WT Hypo mice (Figure 5A,B).

In the heart, PPARα and AMPK activation have also been shown to upregulate FA transporter CD36 levels [30]. The content of CD36 protein was significantly lower in the heart in WT Hypo mice than in WT control mice (Figure 5A). SCD1 deficiency significantly increased CD36 content in the hypothyroid heart, suggesting higher rates of FA transport (Figure 5B). These results indicate that THs affect FA oxidation and transport in the heart, depending on the expression of SCD1.

### 2.7. Inhibition of Deiodinases Increases TG Accumulation in HL-1 Cardiomyocytes

**Figure 6:**
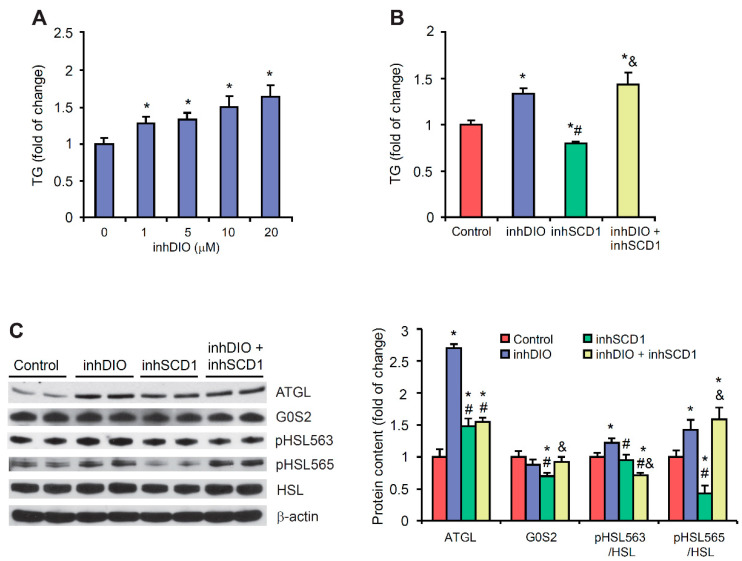
(**A**,**B**) Effect of DIO inhibitor iopanoic acid (inhDIO) and SCD1 inhibitor A939572 (inhSCD1) on triglyceride (TG) content in HL-1 cardiomyocytes. Cells were harvested, and lipid extracts were separated by thin-layer chromatography. The plate was then soaked in a water mixture that contained cupric sulfate and phosphoric acid and charred. Lipids were then quantified by densitometry. (**C**) Protein levels of adipose triglyceride lipase (ATGL), G_0_/G_1_ switch protein 2 (G0S2), hormone-sensitive lipase (HSL), and phosphorylated HSL (pHSL) at Ser563 and Ser565 in HL-1 cardiomyocytes that were treated with the inhDIO and/or inhSCD1. The data are representative of three independent experiments. * *p* < 0.05, vs. control; ^#^ *p* < 0.05, vs. inhDio; ^&^ *p* < 0.05, vs. inhSCD1.

Deiodinases initiate or terminate the actions of THs and thus are critical for the biological effects that are mediated by THs. We used iopanoic acid, a potent pharmacological inhibitor (inhDIO), which inhibits all three DIOs [32], and A939573, a SCD1 inhibitor (inhSCD1) [21], to investigate the relationships between SCD1 and DIO in the regulation of lipid metabolism in cardiomyocytes in vitro. The treatment of HL-1 cells with different concentrations of inhDIO dose-dependently elevated TG content (Figure 6A). Consistent with the results that were obtained in the animal models, SCD1 inhibition in HL-1 cardiomyocytes decreased TG content compared with the control group. Interestingly, the co-treatment of HL-1 cells with inhDIO and inhSCD1 increased TG level compared with untreated HL-1 cells and cells that were treated only with inhSCD1 (Figure 6B).

Next, we tested the hypothesis that changes in lipolysis are involved in the mechanism by which DIO and SCD1 inhibition increase cardiomyocyte steatosis. We measured protein levels of ATGL, G0S2, and HSL and the phosphorylation of HSL. In the present study, DIO inhibition increased ATGL protein levels (>2.5-fold) in HL-1 cardiomyocytes. The simultaneous inhibition of SCD1 reversed this effect (Figure 6C). Moreover, inhSCD1 treatment decreased G0S2 protein levels, whereas incubation of the cells with both inhSCD1 and inhDIO significantly increased G0S2 levels compared with inhSCD1 cells, indicating the repression of ATGL and lipolysis (Figure 6C).

InhDIO increased HSL phosphorylation at Ser563 and Ser565 in HL-1 cardiomyocytes (Figure 6C). SCD1 inhibition did not affect HSL phosphorylation at Ser563, but phosphorylation at Ser565 significantly decreased compared with control and inhDIO-treated HL-1 cells (Figure 6C). Interestingly, incubation of the cells with both inhDIO and inhSCD1 significantly decreased the pHSL(Ser563)/HSL ratio and increased the pHSL(Ser565)/HSL ratio compared with control and inhSCD1-treated HL-1 cells, indicating a decrease in the enzymatic activity of HSL (Figure 6C). These results suggest that an inhibition of DIO activity is a possible mechanism by which lipolysis declines under conditions of SCD1 inhibition, probably accounting for TG accumulation in cardiomyocytes in vitro. However, understanding the relationship between DIO and SCD1 deficiency in vivo requires further studies.

## 3. Discussion

Previously we showed that SCD1 ablation reduces cardiac lipid content in WT mice [19], PPARα-deficient mice [21], and leptin-deficient ob/ob mice [20]. In the present study, we observed an increase in TG and DAG accumulation in the heart in SCD1^−/−^ mice under hyperthyroid conditions compared with SCD1^−/−^ control mice. Importantly, the range of TG accumulation was much greater under conditions of hypothyroidism in the heart in SCD1^−/−^ mice than in the WT mice, clearly indicating that the SCD1 outcome in the heart depends on THs. To our knowledge, this is the first experimental demonstration that SCD1 deficiency leads to an increase in adiposity and the accumulation of lipids in cardiomyocytes.

Our research showed that SCD1 deficiency affected plasma TH levels and TH-dependent signaling pathways in cardiomyocytes. We found that TSH levels decreased, whereas fT3 levels were similar in plasma in SCD1^−/−^ mice compared with WT mice. This condition can be defined as subclinical hyperthyroidism, which is associated with low TSH content and normal fT4 and/or fT3 content [33]. Next, we found that SCD1 deficiency decreased cellular TH transport (through MCT8) and affected TH turnover (i.e., a decrease in *Dio1*, *Dio2*, and *Dio3* expression) and action (i.e., an increase in *Thra* and *Thrb* expression) in cardiomyocytes. Subclinical hyperthyroidism is often associated with metabolic changes, which were observed in SCD1^−/−^ mice (e.g., an increase in energy expenditure, an upregulation of basal thermogenesis, a reduction of body adiposity, and an increase in insulin sensitivity; [17]). Thus, it is possible that alterations of TH metabolism and actions through genomic and non-genomic pathways are involved in the upregulation of metabolism in SCD1-deficient mice.

SCD1 inhibition downregulates the expression of lipogenic genes in HL-1 cells and in the PPARα^−/−^ mouse cardiomyocytes [21]. In contrast, THs generally increased the expression of genes that are involved in de novo lipogenesis, and contradictory FA synthesis in the liver in hypothyroid rats was 3- to 5-fold lower than in euthyroid rats [34]. Interestingly, in hypothyroidism, the loss of *Scd1* expression increased levels of proteins that are involved in lipogenesis in cardiomyocytes, whereas an opposite effect was observed in WT mice. The exception was GPAT1 protein, the content of which decreased in both WT and SCD1^−/−^ mice under conditions of hypothyroidism compared with respective control mice, which is likely related to the function of this protein. GPAT1 is located on the outer mitochondrial membrane where it competes with carnitine palmitoyltransferase 1 for acyl-CoAs, which can enter either the catabolic β-oxidation pathway or the glycerolipid biosynthetic pathway [35]. Thus, in the case of WT Hypo mice, the lower content of GPAT1 can be affected by lower lipogenesis. In SCD1^−/−^ Hypo mice, a reduction of GPAT1 protein can promote higher oxidation. Moreover, GPAT1 contributes to only 10% of total GPAT activity in every tissue except the liver, where it contributes to up to 50% of total GPAT activity [35]. Therefore, lower GPAT1 protein content likely did not affect the increase in lipogenesis in SCD1^−/−^ cardiomyocytes in the hyperthyroid state.

TSH concentrations were nearly 5-times higher in SCD1^−/−^ Hypo mice than in WT Hypo mice. Higher levels of TSH might promote the development of hepatic steatosis by stimulating lipogenesis by binding TSH to TSH receptors to induce SREBP1c [36]. Thus, the activation of lipogenesis in SCD1^−/−^ Hypo mice could be affected by the actions of TSH. Furthermore, the lipogenic effect of hypothyroidism in the SCD1-deficient heart could be mediated by TRβ. Mutant knock-in mice with a targeted mutation of the *Thrb* gene are characterized by excess lipid accumulation in the liver and higher lipogenic enzyme expression [37]. The decrease in lipogenesis in SCD1-deficient cardiomyocytes was accompanied by higher *Thrb* expression, and the activation of this process in the heart in SCD1^−/−^ Hypo mice may be affected by the downregulation of TRβ.

Recent studies underscore the important role of ATGL, the ATGL coinhibitor G0S2, and the ATGL activator ABDH5 in the regulation of heart metabolism [28]. We previously showed that SCD1 ablation/inhibition activated lipolysis in cardiomyocytes [21]. In the present study, although lipolysis was inhibited in the heart in WT Hypo mice, it was still activated in SCD1^−/−^ Hypo mice, reflected by a decrease in G0S2 protein levels and increase in ABDH5 protein levels, leading to the activation of lipolysis by either unblocking or inducing ATGL activity. The increase in lipolysis in SCD1^−/−^ Hypo mice was also associated with HSL activation, reflected by significant increases in the pHSL(Ser563)/HSL ratio and *Lipe* expression. Interestingly, HSL phosphorylation at Ser565 was also elevated in SCD1-deficient cardiomyocytes in hypothyroidism as AMPK was activated, but this elevation was probably insufficient to block HSL phosphorylation at Ser563 by PKA. Phosphorylation of HSL by PKA and PKA translocation to lipid droplets are crucial for lipolysis [29,38]. Moreover, AMPK activation after epinephrine stimulation did not inhibit HSL activity [39].

An opposite effect of hypothyroidism on lipolysis was observed in WT mice, in which lower levels of ATGL, ABDH5, and HSL expression were observed, whereas G0S2 protein levels increased. These results are consistent with findings that hypothyroidism is associated with a decrease in lipolysis in both animals and humans, which can be recovered with TH replacement therapy [40]. Similar to SCD1*^−/−^* mice, both the pHSL(Ser563)/HSL and pHSL(Ser565)/HSL ratios increased in WT Hypo mice, although AMPK phosphorylation decreased. This indicates the involvement of phosphatase A2 inhibition because this enzyme is the most active phosphatase against Ser565. One of the special features of HSL that distinguishes it from most other lipases is that its activity against TG and DAG substrates is controlled by reversible phosphorylation [41].

In the heart, PPARα activation induces the expression of genes that are engaged in nearly every step of the FA utilization pathway [30]. The rate of mitochondrial FA oxidation was lower in the heart in SCD1^−/−^ mice compared with WT controls, which was mainly attributable to a decrease in activity of PPARα and its regulatory pathways [19,20] Interestingly, SCD1 deficiency increased protein levels of PPARα and its coactivator PGC1α in hypothyroidism. An opposite effect was observed in WT mice. PPARα activity is regulated by its association with TRs. Interactions between TRs and PPAR are particularly important in the regulation of lipid metabolism. TRα is the major TR in the heart and crucial for heart rate and cardiac contractility and relaxation [1]. The role of TRα in the regulation of cardiac lipid metabolism is unknown, but the interaction between unliganded TRα and PPARα inhibits PPARα signaling in the liver [42]. Therefore, the decrease in TRα protein content that was observed in SCD1^−/−^ Hypo mice may result in lower TRα availability to bind PPARα, which may then activate this transcription factor.

**Figure 7:**
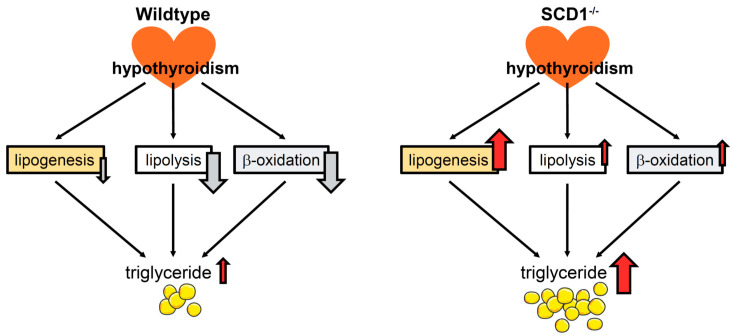
Hypothyroidism leads to the accumulation of lipids in the heart in SCD1^−/−^ and wildtype (WT) mice by regulating different pathways. In WT hypothyroid mice, lipogenesis decreased, but lipid catabolism was also strongly downregulated, which leads to steatosis in cardiomyocytes. In contrast, in SCD1^−/−^ hypothyroid mice, cardiac steatosis is caused by an increase in lipogenesis, despite an upregulation of lipolysis and fatty acid oxidation.

AMPK is important for the regulation of metabolic pathways, such as FA oxidation, glucose uptake, and glycolysis [31]. Treatment with THs increases AMPK phosphorylation in muscle tissues [43,44]. SCD1 deficiency activates AMPK in the liver and skeletal muscles [45,46]. However, in the heart, AMPK phosphorylation and protein levels were unaffected by SCD1 ablation [19]. Interestingly, in the present study, the loss of SCD1 increased the phosphorylation of AMPK and its downstream target ACC in hypothyroidism, whereas a decrease in the phosphorylation of AMPK and ACC was observed in WT Hypo mice. The AMPK pathway was also activated in muscle tissue, WAT, and the hypothalamus in hypothyroid rats [47]. The mechanism of AMPK activation in the heart in SCD1^−/−^ Hypo mice is unknown, but non-genomic pathways (e.g., the Ca2+/calmodulin-dependent protein kinase-β [CaMKKβ] pathway) are involved in AMPK regulation by THs [43]. Overall, these results indicate that SCD1 deficiency increases FA uptake, in which PPARα and AMPK activation upregulate the FA transporter CD36 [30], and oxidation in the heart in hypothyroidism. Hypothyroidism reduced lipid utilization in the heart in WT mice. The possible mechanisms that lead to lipid accumulation in hypothyroidism in SCD1^−/−^ and WT mice are presented in Figure 7.

Both clinical and subclinical hypothyroidism initiate cardiovascular disease through the disturbance of healthy endothelial function by stimulating inflammation and inducing lipid disorders and oxidative stress [48]. The present results further confirmed this assertion, in which levels of inflammatory molecules were elevated in the heart in WT Hypo mice. The role of SCD1 in inflammation appears to vary by tissue type. In many instances, SCD1 inhibition increases inflammation [49]. The overexpression of SCD1 prevented the inflammatory and endoplasmic reticulum stress response to palmitate treatment in primary human myotubes [50] and human arterial endothelial cells [51]. The present study showed that the loss of SCD1 expression in the heart increased the content of proteins that are involved in inflammatory processes. This suggests greater inflammation in the SCD1-deficient myocardium. Interestingly, SCD1 deficiency decreased the content of inflammatory factors, indicating that SCD1 downregulation may exert a protective effect on the heart under hyperthyroid conditions. SCD1 deficiency was shown to lead to saturated FA accumulation, and saturated FAs are the major inducers of apoptosis and inflammatory cytokines in various cell types [18]. Therefore, an increase in the content of inflammatory factors should be expected in the heart in SCD1^−/−^ Hypo mice. A possible explanation for the antiinflammatory effect of SCD1 deficiency in hypothyroidism was the increase in the esterification of FAs through TG synthesis and the attenuation of FFA and DAG accumulation, thus protecting cells against FA-induced cellular toxicity [52]. In the heart in WT Hypo mice, FFA and DAG levels were much higher than in SCD1^−/−^ Hypo mice, possibly contributing to an increase in inflammatory molecule expression. Another possibility is the activation of AMPK and PPARα by SCD1 deficiency in hypothyroidism. Both of these factors negatively regulate proinflammatory signaling pathways, which has been demonstrated in rodent models of systemic inflammation and atherosclerosis [53,54]. However, further research is necessary to clarify this phenomenon.

Evidence indicates that cytokines and other inflammatory factors contribute to the development of abnormal lipid metabolism [55]. LIF was shown to inhibit TG accumulation during adipogenesis, and chronic treatment with LIF decreased protein levels of SREBP1 and FAS in adipocytes [56]. Furthermore, accumulating evidence connects MMP-2 activity with the modulation of inflammation and lipid metabolism in the heart and in noncardiac organs, including the liver and adipose tissue [57]. Fetuin A content was increased in non-alcoholic hepatic steatosis, and the hepatic expression of fetuin A correlated with key enzymes in lipid metabolism [58]. These data suggest the possible involvement of these proinflammatory factors in the development of cardiomyocyte steatosis in SCD1^−/−^ Hypo mice.

In the present study, the link between SCD1 and TH pathways in the regulation of lipid metabolism in cardiomyocytes was also demonstrated in the in vitro model. Using HL-1 cells, we found that the inhibition of DIO activity by iopanoic acid significantly increased TG content in cardiomyocytes that exhibited SCD1 activity inhibition. This process was associated with the inhibition of lipolysis, which is regulated by T3. DIO1 and DIO2 produce T3 from T4 and thus exert an indirect effect on the regulation of metabolic processes [3]. The direct effect of DIO on lipid metabolism was also demonstrated. The disruption of TH activation in Dio2 knockout mice caused obesity, glucose intolerance, and liver steatosis under conditions of thermoneutrality but not at ambient temperature [59]. However, unknown is whether this steatotic effect in HL-1 cardiomyocytes that exhibit SCD1 activity inhibition is related to the direct inhibition of DIO or whether it is caused by a reduction of T3 in the cells. This issue requires further research but highlights the close relationship between SCD1 and THs in the regulation of lipid metabolism in cardiomyocytes.

## 4. Materials and Methods

### 4.1. Materials

ABDH5 (36A, sc-100468), AMPK (H-300, sc-25792), CD36 (sc-9154), DGAT1 (sc-271934), DGAT2 (4C1, sc-293211), FAS (H-300, sc-20140), G0S2 (G-12, sc-133423), GPAT1 (D-10, sc-398135), HSL (G-7, sc-74489), PPARα (H-2, sc-398394), SCD1 (E-15, sc-14720), SREBP1 (2A4, sc-13551), and β-actin (C4, sc-47778) antibodies were obtained from Santa Cruz Biotechnology (Santa Cruz, CA, USA). AKT (C73H10, #2938), ATGL (#2138), ACC (#3662) ERK1/2 (L34F12, #4696), GSK3 (D75D3, #5676), mTOR (7C10, #2983), phosphorylated AKT at Ser473 (pAKT[Ser473]) (#9271), pAKT at Thr308 (pAkt[Thr308]) (#9275), phosphorylated AMPK at Thr172 (pAMPK) (#2531), phosphorylated ERK1/2 at Thr202/Tyr204 (pERK1/2) (197G2, #4377), phosphorylated GSK3 at Ser21/9 (pGSK3) (D17D2, #8566), phosphorylated HSL at Ser563 (pHSL[Ser563]) (#4139), phosphorylated HSL at Ser565 (pHSL[Ser565]) (#4137), phosphorylated mTOR at Ser2448 (pmTOR) (D9C2, #5536), S6K (#9202), and phosphorylated S6K at Thr389 (pS6K) (#9205) antibodies were obtained from Cell Signaling Technology (Hartsfordshire, UK). DIO2 (ab77779), DIO3 (ab102926), PGC1α (ab54481), TRα (ab53729), and TRβ (ab180612) antibodies were obtained from Abcam (Cambridge, UK). GAPDH (6C5, #MAB374) and phosphorylated ACC at Ser79 (pACC) (#07-303) antibodies were obtained from Merck (Darmstadt, Germany). The other chemicals were purchased from Sigma (St. Louis, MO, USA) unless otherwise specified.

### 4.2. Animals

The generation of SCD1^−/−^ knockout mice was previously described [60]. Male wildtype C57/BL6 mice and SCD1^−/−^ mice on a B6 background at 10 weeks of age were fed for 7 weeks a low-iodine diet supplemented with 0.15% propylthiouracil (Ssniff, catalog no. S8435-E060) to induce hypothyroidism [61] or standard laboratory chow (Ssniff, catalog no. V153x). The animals were housed in a pathogen-free facility at room temperature under a 12 h/12 h light/dark cycle. All of the animals were allowed ad libitum access to water and food. The animals were euthanized at 17 weeks of age. All of the protocols that were used in this study were approved by the First Local Ethical Committee for Animal Experiments in Warsaw (permit no. 697/2015, approved 25/03/2015).

### 4.3. Blood and Tissue Sampling

The mice were fasted for 16 h and sacrificed. Blood was collected aseptically by direct cardiac puncture and centrifuged at 3000× *g* at 4 °C for 5 min to collect plasma (samples were aliquoted and stored at −80 °C). The left ventricle of the heart was excised, weighed, frozen in liquid nitrogen, and stored at −80 °C. Epididymal WAT was excised and weighed to determine adiposity of the mice after the diet intervention.

### 4.4. Plasma Lipid, TH, and Glucose Concentrations

Plasma TG levels were measured using a commercial kit (BioSystems, Barcelona, Spain). Plasma FFA levels were measured using the NEFA-HR(2) Kit (Wako, Richmond, VA, USA). Thyroid hormone levels were determined using commercial kits (TSH using the Thyroid Stimulating Hormone kit, Cloud-Clone, Houston, TX, USA; fT3 using the Mouse Free Tri-iodothyronine [Free-T3] enzyme-linked immunosorbent assay [ELISA] kit, CUSABIO, Wuhan, China; T4 using the Mouse/Rat Thyroxine [T4] ELISA kit, Calbiotech, Spring Valley, CA, USA) according to the manufacturers’ instructions. Glucose levels were measured in blood samples that were collected from the tails using glucose strips with an Optium Xido glucose meter (Abbott, Alameda, CA, USA).

### 4.5. Inflammatory Factors

For the detection of cytokines and chemokines in the left ventricles, the Proteome Profiler Mouse Cytokine Array kit (R&D Systems, Minneapolis, MN, USA) was used. The array was performed according to the manufacturer’s instructions. The results were analyzed using Adobe Photoshop software and are expressed as fold changes relative to appropriate controls.

### 4.6. Gene Expression Analysis

Total RNA was isolated from the left ventricle using Total RNA Mini Plus (A&A Biotechnology, Gdynia, Poland) according to the manufacturer’s instructions. DNase-treated RNA (A&A Biotechnology, Gdynia, Poland) was reverse-transcribed using the RevertAid H Minus First Stand cDNA Synthesis Kit (Thermo Scientific, Pittsburgh, PA, USA). Real-time quantitative PCR was performed using the CFX Connect Real-Time PCR Detection System (Bio-Rad, Hercules, CA, USA). SsoAdv Univer SYBR SMX (Bio-Rad, Hercules, CA, USA) was used to detect and quantify mRNA expression. The relative expression of each sample was determined after normalization to β-actin or 60S ribosomal protein L32 (RPL32) using the ΔΔCt method. A list of primers for real-time PCR is presented in Table 2.

### 4.7. Culture of HL-1 Cardiomyocytes

HL-1 cardiomyocytes were obtained from W.C. Claycomb (Louisiana State University, New Orleans, LA, USA). The cells were cultured on a gelatin (0.02% [*w*/*v*])/fibronectin (10 μg/mL) matrix and maintained in Claycomb medium supplemented with 10% (*v*/*v*) fetal bovine serum, 2 mM/L glutamine, 0.1 mM/L norepinephrine, 100 U/mL penicillin, and 100 U/mL streptomycin in a 5% CO_2_ atmosphere at 37 °C [62]. The medium was changed every 24 h. To inhibit SCD1 and DIO activity, the cells were incubated with 2 μM of the SCD1 inhibitor A939572 (inhSCD1; Biofine International, Blain, WA, USA) and/or 10 µM iopanoic acid (inhDIO; Sigma) in Claycomb medium with a decrease to 1% (*v*/*v*) fetal bovine serum for 24 h. Control cells were incubated for the same period of time with 1% (*v*/*v*) fetal bovine serum Claycomb medium supplemented with a corresponding concentration of dimethylsulfoxide and ethanol diluted in PBS for inhSCD1 and inhDIO, respectively.

### 4.8. Western Blot Analysis

HL-1 cells were collected and lysed for 30 min in ice-cold lysis buffer that contained 20 mM Tris-HCl (pH 7.4), 150 mM NaCl, 1 mM ethylenediaminetetraacetic acid (EDTA), 1% Triton X-100, 1 mM dithiothreitol (DTT), 0.1 mM phenylmethane sulfonyl fluoride (PMSF), 1 mM sodium orthovanadate (Na_3_VO_4_), 10 μg/μL leupeptin, 5 μg/μL pepstatin A, and 2 μg/μL aprotinin. For further analyses, whole-cell lysate supernatants that were obtained by centrifugation at 12,000× *g* at 4 °C were used. The left ventricle samples from SCD1^−/−^ and WT mice were lysed in ice-cold lysis buffer that contained 20 mM Tris-HCl (pH 7.4), 2 mM ethylene glycol-bis[β-aminoethyl ether]-*N*,*N*,*N′*,*N′*-tetraacetic acid (EGTA), 2 mM EDTA, 2 mM Na_3_VO_4_, 1 mM PMSF, 10 mM β-mercaptoethanol, 10 μg/μL leupeptin, 5 μg/μL pepstatin A, and 2 μg/μL aprotinin and centrifuged at 10,000× *g* for 20 min at 4 °C. The protein content in the lysates was determined using the Bio-Rad Protein Assay (Bio-Rad) with bovine serum albumin as the reference. The proteins samples were separated on 10% sodium dodecyl sulfate-polyacrylamide gel electrophoresis gels and transferred to polyvinylidene difluoride membranes (Millipore, Billerica, MA, USA). Western blot analysis was performed using appropriate antibodies. The proteins were visualized using SuperSignal West Pico PLUS Chemiluminescent Substrate (Thermo Scientific) and quantified by densitometry. Protein levels are expressed relative to the abundance of GAPDH or β-actin. Phosphorylated protein levels are expressed relative to the abundance of the unphosphorylated isoform of the respective protein.

### 4.9. Measurement of Lipids

Lipids were extracted from HL-1 cardiomyocytes and the left ventricles according to the method of Bilgh and Dyer [63] and quantified as described previously [21]. Extracted lipids were separated by thin-layer chromatography on silica gel 60 plates (Merck, Darmstadt, Germany) in heptane/isopropyl ether/glacial acetic acid (60/40/4 [*v*/*v*/*v*]) with authentic standards. To visualize lipid bands, the plate was soaked in a water mixture that contained 10% cupric sulfate and 8% phosphoric acid and then burned in at 140 °C for 20 min. The lipids were then quantified by densitometry.

### 4.10. Statistical Analysis

The data are expressed as mean ± SD, with *n* = 5 mice/group. The data that were obtained using HL-1 cells are representative of three independent experiments. Multiple comparisons were performed using one-way analysis of variance (ANOVA) followed by Tukey’s post hoc test using Prism 8.3.0 software (GraphPad, La Jolla, CA, USA). A two-sided *t*-test was applied when differences between two groups were analyzed. The level of significance was *p* < 0.05.

## Figures and Tables

**Table 1:** Adiposity, heart weight, concentrations of plasma TG, FFA, glucose, TSH, fT3, and T4 in WT, WT Hypo, SCD1^−/−^, and SCD1^−/−^ Hypo mice.

	WT	WT Hypo	SCD1^−/−^	SCD1^−/−^ Hypo
WAT/BW (mg/g)	11.6 ± 0.7	15.0 ± 0.7 *	6.6 ± 0.6 *^,#^	10.8 ± 0.8 ^#,&^
HW/BW (mg/g)	6.0 ± 0.2	4.5 ± 0.1 *	6.3 ± 0.3 ^#^	4.7 ± 0.1 *^,&^
TG (mg/dl)	85.5 ± 7.7	44.5 ± 3.4 *	55.5 ± 7.2 *	54.8 ± 7.8 *
FFA (mg/dl)	23.8 ± 2.2	18.7 ± 1.8 *	21.7 ± 2.2	23.4 ± 3.1
Glucose (mg/dl)	118.9 ± 14.9	159.5 ± 19.7 *	121.4 ± 4.7	163.2 ± 12.5 *^,&^
TSH (pg/mL)	0.104 ± 0.005	0.21 ± 0.07 *	0.066 ± 0.02 *^,#^	0.948 ± 0.61 *^,#,&^
fT3 (pMol/dl)	6.8 ± 0.5	6.9 ± 0.5	6.3 ± 0.8	6.9 ± 0.7
T4 (mg/dl)	2.1 ± 0.2	0.4 ± 0.1 *	1.4 ± 0.2 *^,#^	0.5 ± 0.2 *^,&^

*n* = 10. BW—body weight, HW—heart weight, WAT—white adipose tissue, TG—triglyceride, FFA—free fatty acid, TSH—thyroid stimulate hormone, fT3—free triiodothyonine, T4—thyroxine, Hypo—hypothyroid. * *p* < 0.05 vs. WT; ^#^ *p* < 0.05 vs. WT Hypo; ^&^ *p* < 0.05 vs. SCD1^−/−^.

**Table 2:** Real-time PCR primers list.

Gene	Forward Primer	Reverse Primer
*Abhd5*	TGGTGTCCCACATCTACATCA	CAGCGTCCATATTCTGTTTCCA
*Dio1*	GCTGAAGCGGCTTGTGATATT	GTTGTCAGGGGCGAATCGG
*Dio2*	AATTATGCCTCGGAGAAGACCG	GGCAGTTGCCTAGTGAAAGGT
*Dio3*	CACGGCCTTCATGCTCTGG	CGGTTGTCGTCTGATACGCA
*Lipe*	TTCTCCAAAGCACCTAGCCAA	TGTGGAAAACTAAGGGCTTGTTG
*Pnpla2*	CAACGCCACTCACATCTACGG	GGACACCTCAATAATGTTGGCAC
*Rpl32*	AGTTCCTGGTCCACAATGTCA	GCACACAAGCCATCTACTCATT
*Slc16a2*	GTGCTCTTGGTGTGCATTGG	CCGAAGTCCCCGGCATAGG
*Thra*	GGTCACCAGATGGAAAGCGAA	CCTTGTCCCCACACACGAC
*Thrb*	ACACCAGCAATTACCAGAGTG	GCAGCTCGAAGGGACATGA
*β-actin*	TTCTTGGGTATGGAATCCTGT	AGCACTGTGTTGGCATAGAG

## Data Availability

The data presented in this study are available on request from the corresponding author.

## Abbreviations

ABDH5	α/β-hydrolase domain containing 5
AKT	protein kinase B
AMPK	5′-adenosine monophosphate-activated protein kinase
ATGL	adipose triglyceride lipase
DAG	diacylglycerol
DGAT	diacylglycerol acyltransferase
DIO	deiodinase
ERK1/2	extracellular signal-regulated kinases 1/2
FA	fatty acids
FFA	free fatty acids
fT3	free triiodothyronine
G0S2	G0/G1 switch protein 2
GPAT	glycerol-3-phosphate acyltransferase
GSK3	glycogen synthase kinase 3
HSL	hormone-sensitive lipase
IGFBP-2	insulin-like growth factor-binding protein-2
inhDIO	deiodinases inhibitor (iopanoic acid)
inhSCD1	stetaroyl-CoA desaturase 1 inhibitor (A939573)
LIF	leukemia inhibitory factor
LIX	C-X-C motif chemokine 5
MCT8	monocarboxylate transporter 8
MMP-2	matrix metalloproteinase 2
mTOR	mammalian target of rapamycin
PGC1α	peroxisome proliferator-activated receptor gamma coactivator 1α
PPARα	peroxisome proliferator-activated receptor α
S6K	S6 kinase
SCD1	stearoyl-CoA desaturase 1
SREBP1	sterol regulatory element-binding protein 1
T3	triiodothyronine
T4	thyroxine
TG	triglyceride
TH	thyroid hormones
TNFα	tumor necrosis factor α
TR	thyroid receptor
TSH	thyroid stimulate hormone
VCAM-1	vascular cell adhesion protein 1
WT	wildtype mice
